# Satisfied with Life? The Protective Function of Life Satisfaction in the Relationship between Perceived Stress and Negative Mental Health Outcomes

**DOI:** 10.3390/ijerph20186777

**Published:** 2023-09-18

**Authors:** Anita Padmanabhanunni, Tyrone B. Pretorius, Serena Ann Isaacs

**Affiliations:** Department of Psychology, University of the Western Cape, Bellville 7530, South Africa; apadmana@uwc.ac.za (A.P.); sisaacs@uwc.ac.za (S.A.I.)

**Keywords:** anxiety, depression, hopelessness, life satisfaction, mediation, moderation, protective factors

## Abstract

Life satisfaction is a key index of well-being, yet few studies have examined its role as a protective factor in the context of the COVID-19 pandemic. The current study expands the research in this area through an examination of the role of life satisfaction in the relationship between perceived stress and negative indices of mental health. Participants were university students (N = 322) who completed the Perceived Stress Scale, the Satisfaction with Life Scale, and short forms of the trait scale of the Spielberger State-Trait Anxiety Inventory, the Center for Epidemiological Depression Scale, and the Beck Hopelessness Scale. The results indicate that life satisfaction had a health-sustaining effect on indices of well-being. It also moderated the relationship between perceived stress, on the one hand, and anxiety and hopelessness, on the other hand. Further, life satisfaction played a partial mediating role in the relationship between perceived stress and indices of mental health. The findings suggest that life satisfaction could be a protective factor in the context of stressful life events. Cultivating life satisfaction through mindfulness training and the enhancement of gratitude could potentially sustain mental health.

## 1. Introduction

The field of positive psychology has spurred research and interest in psychological well-being. Life satisfaction represents a central indicator of subjective well-being and mental health. It is a multidimensional construct and although there is no single comprehensive theory of life satisfaction, theorists distinguish between top-down and bottom-up conceptualizations [1]. The former conceptual framework views life satisfaction as a function of stable personality traits and suggests that certain people are predisposed to feel a greater sense of satisfaction with their lives [1]. According to top-down models of well-being, various dispositional factors influence global appraisals or judgements of life satisfaction. These appraisals, in turn, influence satisfaction with various life domains. Support for this view comes from a meta-analysis of 249 studies [2], which concluded that the Big Five personality traits accounted for 18% of the variance in life satisfaction. A more recent study [3] found that personality traits explained 14.8% of the variance in total life satisfaction suggesting that personality may not fully account for satisfaction with life. The bottom-up approach views life satisfaction as a function of one’s satisfaction with various life domains including work, leisure, family, friends, and health [1]. Life satisfaction is not considered to be an average of domain satisfaction as people tend to appraise each domain differently. Instead, satisfaction with domains that correspond with the individual’s values has been associated with overall life satisfaction [1]. Certain theorists (e.g., [3]) have highlighted the need for an integrated approach to life satisfaction that considers the influence of dispositional characteristics, as well as the satisfaction with domains that correspond with the individual’s values. 

Existing research [4,5] has examined the correlates and predictors of life satisfaction and confirmed that levels of well-being are higher among individuals with a higher socioeconomic status, social support, a significant partner relationship, financial resources, good health, and specific personality characteristics (e.g., low neuroticism) than among their peers. Cross-cultural differences in life satisfaction have also been reported. For example, Oishi and Colleagues [6] found that satisfaction with esteem-related needs (e.g., freedom) predicted global life satisfaction among those from individualistic cultures compared to those from collectivist cultures. Relationship harmony was more likely to predict life satisfaction among those belonging to collectivistic cultures [6]. Nevertheless, health and socio-economic status have been consistently identified as the most salient factors associated with life satisfaction and subjective well-being [7].

Mental health problems constitute a salient category of predictors of life satisfaction [8]. The COVID-19 outbreak and the measures implemented to curb the spread of the virus have significantly disrupted daily life and contributed to significant psychological distress for many individuals. Various systematic reviews and meta-analytic studies have reported worldwide increases in depression, anxiety, loneliness, hopelessness, and post-traumatic stress disorder (PTSD). A meta-analytic study and systematic review of mental health prior to and during the disease outbreak [9] highlighted significant increases in depressive symptoms in the context of the pandemic. Similarly, Cénat and colleagues [10] found increases in depression, anxiety, PTSD, and loneliness in select countries (e.g., United States and Latin American countries), while Dragioti and Colleagues [11] found elevated rates of PTSD among people infected with COVID-19, as well as depression, insomnia, anxiety, and suicidal ideation. These mental health conditions were found to be more common in the context of low-to-middle-income countries. 

COVID-19-related mental health difficulties have been reported to impact life satisfaction among different groups in distinctive ways. For example, a Vietnamese study [12] among university students found that anxiety and fear related to COVID-19 were positively associated with life satisfaction. In other words, students who reported greater fear and anxiety also experienced greater life satisfaction than their peers. This finding was ascribed to the pandemic possibly leading individuals to review their personal values and prioritize aspects of their lives that they found meaningful (e.g., personal relationships, work–life balance, etc.). The study also found that high levels of psychological distress negatively impacted life satisfaction. Lopes and colleagues [13] investigated predictors of life satisfaction among Brazilian university students and reported that stress, anxiety, and depressed mood were negatively correlated with life satisfaction. Female gender, substance use, and pre-existing comorbid conditions were also found to be related to psychological distress. The researchers hypothesized that pandemic-related restrictions on in-person contact increased loneliness among students and aggravated stress levels. A study of the German population undertaken a year after the disease outbreak [14] reported a decline in mental health. Depression and loneliness levels were found to have increased, along with significant decreases in life satisfaction. Vulnerable groups—including young adults and individuals with a history of mental health disorders—displayed increased levels of distress overall than their peers. 

Although research has confirmed that the pandemic was associated with significant mental health disorders and psychological distress, many studies (e.g., [15]) have also found that a significant portion of the population were able to effectively cope and adapt to adverse conditions, which suggests the presence of protective factors. Researchers have found significant support for the stress-buffering hypothesis, which proposes that protective factors can potentially buffer the influence of adverse events and stressors [16]. For example, a Portuguese study [17] reported that life satisfaction mediated the association between depression and burnout, and between anxiety and burnout, among nurses and appeared to be a substantive protective factor in psychological health. A South African study [18] reported that increased adaptive cognitive appraisals were related to reduced feelings of hopelessness and anxiety among young adults. Adaptive cognitive appraisals were highlighted as a salient protective resource for promoting mental health during the pandemic. Shug and colleagues [19] found that psychosocial resources, including social support and optimism, protected against depression and generalized anxiety among German health care workers. A Chinese study [20] reported that mindfulness and perceived social support were protective factors against anxiety and depression among university students. In the current study, we aim to extend the knowledge base on protective factors through an examination of the role of life satisfaction in the relationship between perceived stress and negative mental health outcomes among South African students in the context of the COVID-19 outbreak. 

Globally, students enrolled at higher education institutions experienced additional stressors owing to the measures aimed at curbing the spread of COVID-19. The closure of universities, disruption of in-person academic activities, and transition to online education led to uncertainty regarding students’ professional training, as well as stress and anxiety about their future careers. Social-distancing measures limited opportunities for connection and contributed to loneliness among students. As was true of the general population, students experienced fear and anxiety regarding their own risk of contagion and the safety of their families and significant others. Studies undertaken in different countries (e.g., Spain [21]; Brazil [13]; Ethiopia [22]) confirmed increased levels of anxiety, stress, and depression among college students during the disease outbreak. In South Africa, a country with significant socioeconomic disparities, student anxiety and stress were also related to limited access to information communication technology and resources needed to effectively cope with the pandemic (e.g., personal protective equipment), as well as threats to job and food security [23]. The current study expands the knowledge base on the protective function of life satisfaction through an examination of its role in the relationship between perceived stress and adverse mental health outcomes among South African students. In doing so, the study aims to identify salient protective factors and the pathways through which they operate. This type of information may be important for targeted intervention efforts that aim to enhance student well-being and internal capacities to manage adversity. 

The current study focused on the protective role of life satisfaction in the relationship between perceived stress and indices of negative mental health and, accordingly, we examined the following hypotheses:

**H1.**  *Life satisfaction will mediate or moderate the relationship between perceived stress and anxiety.*

**H2.**  *Life satisfaction will mediate or moderate the relationship between perceived stress and hopelessness.*

**H3.**  *Life satisfaction will mediate or moderate the relationship between perceived stress and depression.*

## 2. Materials and Methods

### 2.1. Participants and Procedure

The current study was cross-sectional in nature and undertaken at a South African higher education institution located in the Western Cape Province of the country. A random number generator was used to select a random sample of 1700 students via the office of the registrar of the university. An electronic questionnaire comprising the instruments used in the study was created using the Google Forms platform. The link to the electronic questionnaire was distributed to select students along with an invitation to participate in the study. Reminders to participate were send out bi-weekly. Once the participant clicked on the link, they were directed to a landing page that requested informed consent. Only following the provision of informed consent could the participants proceed to the survey. The survey was anonymous and no personal information was collected. The final student sample consisted of 322 participants (response rate: 18.94%). Most students were women (77%) and lived in an urban area (87.3%). The average age of the students in the sample was 26.01 years (*SD* = 10.19). The study was conducted during March–July 2022, when the COVID-19 disease outbreak was still considered a global pandemic. While no lockdown restrictions were in force in South Africa, the university where the study was conducted still operated remotely. At that stage, 86.6% of the participants confirmed that they had been vaccinated.

### 2.2. Measures

The electronic survey consisted of the following measures, namely: the Satisfaction with Life Scale (SWLS) [24], Perceived Stress Scale (PSS) [25], short versions of the trait scale of the Spielberger State-Trait Anxiety Inventory (STAI-T5) [26], the Beck Hopelessness Scale (BHS-9) [27], and the Center for Epidemiological Depression Scale (CES-D10) [28]. Owing to the questionnaire being lengthy, limited demographic variables were included in the study.

The PSS measures the individual’s appraisals of life events as potentially stressful (i.e., as unpredictable, uncontrollable, and overwhelming). Item examples include “In the last month, how often have you felt that you were on top of things?” and “How often have you felt nervous or stressed?” The respondent rates these items using a five-point Likert scale from “never” (0) to “very often” (4). The authors of the PSS reported internal consistency reliability estimates of 0.84–0.86 [25]. Lee [29] undertook a systematic review of studies using the PSS and reported that the estimates of reliability for all reviewed studies exceeded 0.70. A South African study [30] using the PSS reported similar results (i.e., Cronbach’s alpha of 0.87). Studies have provided evidence for a one-factor structure for the PSS [31,32].

The SWLS intends to assess an individual’s cognitive evaluation of the extent to which they are satisfied with their life as a whole [24]. Item examples are: “The conditions of my life are excellent” and “In most ways, my life is close to my ideal.” The SWLS is a 5-item instrument and is rated using a seven-point Likert scale from “strongly disagree” (1) to “strongly agree” (7). Diener and colleagues reported an internal consistency reliability of 0.82. They demonstrated the validity of the instrument through an evaluation of the relationships between the SWLS and several other measures of subjective well-being [24]. In South Africa, the reliability of the SWLS was found to be satisfactory (*α* = 0.90) and the unidimensional structure of the scale was confirmed [33].

The STAI-T5 is a measure of anxiety and comprises 5 items. It is a short-form version of the 20-item STAI-T [34]. Example items of the STAI-T5 are: “I get in a state of tension or turmoil as I think over my recent concerns and interests” and “I worry too much over something that really doesn’t matter.” The STAI-T5 is rated on a four-point Likert scale from “not at all” (1) to “very much so” (4). Zsido and colleagues reported an internal consistency reliability of 0.82 for STAI-T5. A South African study used both classical test theory and item response theory and confirmed the reliability, validity, and unidimensional structure of the STAI-T5 [35]

The CES-D10 is a measure of the symptoms of depression and consists of 10 items. It is a short-form version of the original 20-item CES-D [36]. Example items of the CES-D10 are: “My sleep was restless” and “I felt that everything I did was an effort.” The instrument is rated on a four-point scale from “rarely or none of the time” (0) to “most or all of the time” (3). Zhang and colleagues reported an alpha coefficient of 0.88 and demonstrated that the CES-D10′s ability to classify participants with depression was comparable to that of the original CES-D [28]. The original CES-D has been used in a South African study on school teachers and the reported alpha coefficient was 0.92 [37]. Thröstur and colleagues used exploratory and confirmatory factor analysis and found that a one-factor structure provided a good fit for the CES-D10 [38].

The BHS-9 was designed to assess a core feature of depression, namely a sense of hopelessness. The BHS-9 represents the 9-item version of the original 20-item BHS [39]. It measures three components of hopelessness, namely future expectations, feelings about the future, and loss of motivation [27]. Example items of the BHS-9 include: “All I can see ahead of me is unpleasantness rather than pleasantness” and “I don’t expect to get what I really want.” The BHS-9 is scored using a “true/false” dichotomous scale. Balsamo and colleagues [27] reported a satisfactory reliability (Mokken scale reliability = 0.87, α = 0.86, latent class reliability coefficient = 0.89) and used the automated item selection procedure in Mokken analyses to confirm the unidimensional structure of the BHS. The original version of the BHS was used in South Africa among a cohort of university students and the internal consistency reliability was satisfactory (α = 0.88) [40].

### 2.3. Ethics

This study received ethical clearance from the institutional review board of the University of the Western Cape (ethics reference number: HS22/2/9, February 2022), and the study adhered to the guidelines of the Declaration of Helsinki. All participants provided informed consent and participated voluntarily. No identifiers were used in the survey. 

### 2.4. Data Analysis

The data analyses for the study was undertaken using IBM SPSS for Windows version 28 (IBM Corp., Armonk, NY, USA). Prior to the analysis relating to the objective of the study, we examined the normality of the data using indices of skewness and kurtosis. It is suggested that data are considered to be normal if the skewness is between −2 to +2 and the kurtosis is between −7 to +7 [41]. The means and standard deviations, reliabilities (Cronbach’s alpha and McDonald’s omega), and intercorrelations between all variables (Pearson’s r) were generated. For the moderation (Model 1) and mediation (Model 4) analyses, we used the PROCESS macro in SPSS [42]. For the moderation analyses, the interaction term was generated using mean-centered variables, and the nature of significant interactions was plotted using the visualization code provided by PROCESS. For both the moderation and mediation analyses, we used the 95% confidence intervals (CIs) to evaluate the significance of effects.

## 3. Results

The descriptive statistics, reliabilities, and intercorrelations between study variables are reported in Table 1.

Table 1 reflects that all of the skewness values were between −2 and +2, while all of the kurtosis values were between −7 and +7, thus indicating that the data were normally distributed. The internal consistency coefficients in Table 1 are satisfactory (alpha and omega: 0.84–0.88). Table 1 also shows that there was a negative association between perceived stress and life satisfaction (r = −0.53, *p* < 0.001). Perceived stress was positively associated with anxiety (r = 0.60, *p* < 0.001), hopelessness (r = 0.47, *p* < 0.001), and depression (r = 0.66, *p* < 0.001). There was a negative association between life satisfaction and the negative indices of mental health (anxiety: r = −0.41, *p* < 0.001; hopelessness: r = −0.52, *p* < 0.001; depression: r = −0.53, *p* < 0.001). The effect size of the associations between perceived stress and hopelessness, life satisfaction and anxiety, and anxiety and hopelessness can be considered moderate. The effect size of all other associations can be considered substantial. Thus, elevated levels of perceived stress were associated with reduced life satisfaction and high levels of hopelessness, anxiety, and depression. Greater levels of life satisfaction were associated with lower anxiety, hopelessness, and depression.

The results of the moderation analyses with life satisfaction as the moderator are presented in Table 2.

**Figure 1:**
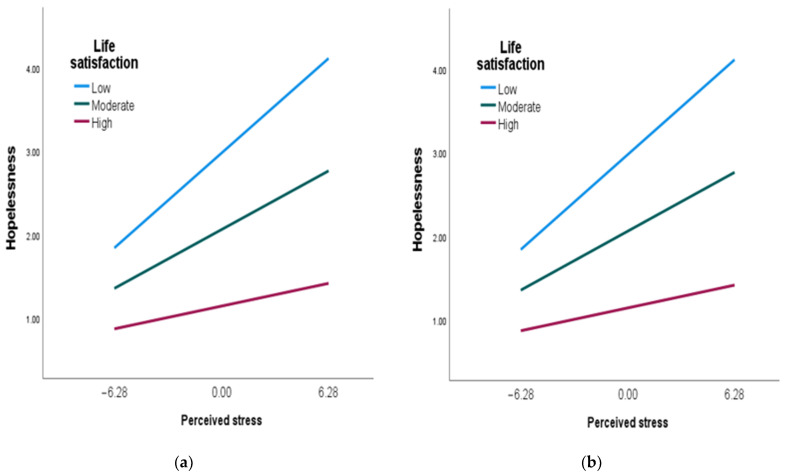
Plot of the interaction between life satisfaction and perceived stress in respect of anxiety and hopelessness: (**a**) anxiety, (**b**) hopelessness.

The zero-order correlation between stress and anxiety in Table 1 was significant (r = 0.60, *p* < 0.001). However, when considered with the moderator of life satisfaction, that relationship was no longer significant (B = 0.141, 95% CI [−0.023, 0.304]). The interaction term of perceived stress and life satisfaction was significant (B = 0.011, 95% CI [0.003, 0.304], which indicates that life satisfaction moderated the perceived stress–anxiety relationship. Similarly, life satisfaction was a moderator of the perceived stress–hopelessness relationship (B = −0.010, 95% CI [−0.014, −0.005]). These two findings support Hypotheses 1 and 2. However, the interaction term of perceived stress and life satisfaction was not significant with regard to depression (B = −0.007, 95% CI [−0.018, 0.004]). The moderating role of life satisfaction with respect to anxiety and hopelessness is visually presented in Figure 1.

Figure 1 shows that participants who reported high levels of life satisfaction demonstrated lower levels of hopelessness and anxiety in the presence of high levels of stress compared to participants who reported moderate and low amounts of life satisfaction. 

**Figure 2:**
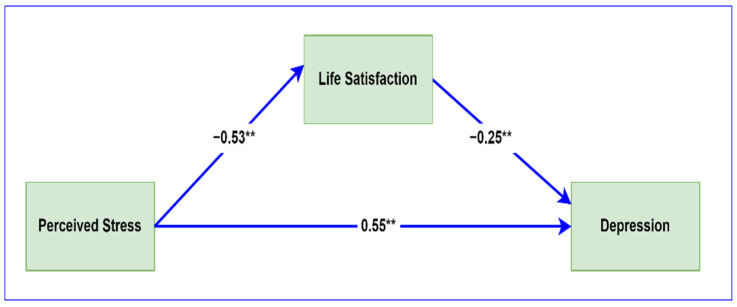
Visual representation of the mediating role of life satisfaction in the stress–depression relationship. Regression coefficients are standardized. ** *p* < 0.001.

Since life satisfaction was not a moderator of the perceived stress–depression relationship, we used PROCESS to examine the potential role of life satisfaction as a mediator in this relationship. The mediation results are presented in Table 3, and a conceptual model of the role of life satisfaction in the stress–depression relationship is shown in Figure 2.

Both the direct (β = 0.526, *p* < 0.001) and indirect (β = 0.135, *p* < 0.001) effects of perceived stress on depression were significant. This finding demonstrates that life satisfaction partially mediated the perceived stress–depression relationship and that the direct effect of life satisfaction on depression was also significant (β = −0.253, *p* < 0.001), and this finding supports Hypothesis 3.

## 4. Discussion

The COVID-19 disease outbreak and related containment measures contributed to increased levels of perceived stress and significantly impacted mental health and well-being globally. However, a significant portion of people were able to cope effectively and experienced minimal disruptions to their health and well-being [12,40]. This variability in response to adversity points to the role of protective factors in promoting coping and adaptation. In the current study, we examined the role of life satisfaction in the relationship between perceived stress and negative indices of mental health. The results demonstrate that life satisfaction plays various roles in this relationship, namely direct, moderating, and mediating.

First, life satisfaction had a direct effect on negative indices of mental health. In the literature, this is referred to as a health-sustaining effect. For example, in a study of social support, Shumaker and Brownell maintain that even in the absence of stress, social support is related to low levels of distress [43]. Thus, even in the absence of stress, life satisfaction positively impacts negative mental health. This result supports prior research reporting a negative relationship between life satisfaction and mental health [7,8,44]. Given that the current study is cross-sectional in nature, it is equally plausible that a high level of negative mental health might impact one’s level of life satisfaction.

Second, life satisfaction moderated the relationship between perceived stress and hopelessness as well as anxiety. Participants with high levels of life satisfaction had lower levels of anxiety and hopelessness compared to participants with low levels of life satisfaction at both elevated and low levels of perceived stress. In the literature, this is referred to as a stress-buffering or stress-reducing effect [43], in the sense that life satisfaction reduces or buffers the impact of perceived stress on mental health. This finding is similar to the findings of previous studies that have reported that life satisfaction moderated the indirect relationship of social-networking site usage and depression [45], as well as between autonomy-granting parenting and children’s depression [46]. Previous research (e.g., [47]) has suggested that individuals’ appraisals that they have a meaningful life, which represents a core component of life satisfaction, are related to reduced stress levels and the use of adaptive coping strategies. The pandemic may have led university students to reflect on their lives, relationships, and academic careers and experience a heightened sense of appreciation for these facets of their lives. This type of reflection can lead to a sense of gratitude and produce positive emotions [48]. The broaden-and-build theory of emotion [49] postulates that experiencing positive emotions can enhance self-confidence and the use of adaptive coping resources in managing stressors and thereby reduce distress [50]. Although the current study did not assess gratitude or social support, it is likely that the threat posed by the COVID-19 outbreak to the well-being of young adults’ family members and significant others led them to value their close relationships and re-prioritize spending time with family and friends. In turn, this may have enhanced their social support base and contributed to increased life satisfaction and lowered levels of distress. 

Third, the results demonstrated that life satisfaction partially mediated the relationship between perceived stress and mental health outcomes. In this regard, life satisfaction is the pathway through which perceived stress impacts mental health. It is plausible that heightened levels of perceived stress impact life satisfaction, which in turn negatively impacts mental health. This finding supports research findings by Tamarit and colleagues pertaining to the mediating role of life satisfaction in the relationship between COVID-19-related worries and depression and COVID-19-related worries and anxiety [51]. The finding can also be explained through cognitive appraisal theory [18]. It is probable that when encountering a perceived stressor, an individual’s appraisals of having a meaningful life may contribute to their ability to view the stressor as an isolated event in the broader context of their lives. This perspective can modify the perceptions of the stressor as overwhelming and intractable and facilitate coping. 

The findings of this study indicate that life satisfaction could be a potential protective resource and sustain mental health during times of crisis. If cultivated in contextually appropriate ways, life satisfaction can reduce the impact of future stressors. Existing interventions for enhancing meaning in life have focused on promoting self-awareness and developing gratitude through mindfulness-based interventions [52]. These types of interventions can assist young adults by developing their capacity for perspective taking and enhance their sense of self-efficacy in negotiating life stressors. 

There are several limitations to the current study. First, owing to the use of an electronic survey, it is probable that those students with an interest in mental health outcomes associated with the pandemic may have been more likely to participate, thus contributing to selection bias. Second, the responses to the survey may have been influenced by social desirability bias and future studies that use a triangulation design may be beneficial to confirm the results. Third, causal inferences need to be undertaken with caution owing to the cross-sectional design of the study. A longitudinal research approach would help to corroborate the findings. Fourth, no retrospective information was collected; therefore, it is likely that some respondents’ mental health challenges preceded the pandemic. Finally, the study participants were predominantly women, which limits the extent to which population-level generalizations can be made. However, women are over-represented in college populations and our results correspond to the existing literature. 

## 5. Conclusions

Although life satisfaction has been extensively investigated during the COVID-19 outbreak, few studies have assessed its role as a protective resource in the association between stress and adverse psychological outcomes. The current study extends the research in this area by examining the direct, moderating, and mediating roles of life satisfaction. The study found that life satisfaction was the pathway through which perceived stress impacted on mental health. Specifically, life satisfaction moderated the relationship between perceived stress, and hopelessness as well as anxiety, and partially mediated the relationship between perceived stress and indices of psychological distress. These findings suggest that life satisfaction could be a potential protective resource and sustainer of psychological well-being in the context of adversity.

## Figures and Tables

**Table 1:** Summary indices: descriptive statistics, reliability values, and intercorrelations.

Variable/Scale	1	2	3	4	5
1. Perceived stress	-				
2. Life satisfaction	−0.53 **	-			
3. Anxiety	0.60 **	−0.41 **	-		
4. Hopelessness	0.47 **	−0.52 **	0.46 **	-	
5. Depression	0.66 **	−0.53 **	0.66 **	0.50 **	-
Mean	23.9	19.35	12.4	2.3	14.15
SD	6.3	7.1	4.1	2.4	6.8
Minimum	6	5	5	0	0
Maximum	39	35	20	9	30
Skewness	−0.18	−0.03	0.03	1.21	0.05
Kurtosis	−0.18	−0.74	−0.88	0.59	−0.73
Alpha	0.85	0.86	0.88	0.84	0.84
Omega	0.86	0.86	0.88	0.84	0.85

Note. Perceived Stress Scale = perceived stress; Satisfaction with Life Scale = life satisfaction; Trait Scale of the State-Trait Anxiety Inventory-5 = anxiety; Beck Hopelessness Scale-5 = hopelessness; Centre for Epidemiological Studies Depression Scale-10 = depression. ** *p* < 0.001.

**Table 2:** Life satisfaction as a moderator of the relationship between perceived stress and mental health.

Variable	Beta	SE	95% CI	*p*
Anxiety as dependent variable				
Perceived stress	0.141	0.083	[−0.023, 0.304]	0.091
Life satisfaction	−0.331	0.096	[−0.520, −0.142]	<0.001
Perceived stress × Life satisfaction	0.011	0.004	[0.003, 0.018]	0.006
Hopelessness as dependent variable				
Perceived stress	0.301	0.050	[0.201, 0.400]	<0.001
Life satisfaction	0.103	0.583	[−0.012, 0.216]	0.079
Perceived stress × Life satisfaction	−0.010	0.002	[−0.014, −0.005]	<0.001
Depression as dependent variable				
Perceived stress	0.571	0.051	[0.470, 0.672]	<0.001
Life satisfaction	−0.242	0.046	[−0.332, −0.152]	<0.001
Perceived stress × Life satisfaction	−0.007	0.006	[−0.018, 0.004]	0.234

Note. Beta = unstandardized coefficient, SE = standard error, CI = confidence interval.

**Table 3:** The mediating role of life satisfaction in the perceived stress–depression relationship.

Effect	Beta	SE	β	95% CI	*p*
Direct effects					
Perceived stress → Depression	0.567	0.051	0.526	[0.47, 0.67]	<0.001
Life satisfaction → Depression	−0.243	0.046	−0.253	[−0.33,−0.15]	<0.001
Indirect effects					
Perceived stress → Life Satisfaction → Depression	0.145	0.033	0.135	[0.08, 0.21]	<0.001

Note. Beta = unstandardized coefficient, β = standardized coefficient, CI = confidence interval.

## Data Availability

The data that support the findings of this study are available from the corresponding author upon reasonable request.
